# Bis{μ-2-[(dimethyl­amino)­meth­yl]benzene­tellurolato}bis­[chlorido­palladium(II)] dichloro­methane hemisolvate

**DOI:** 10.1107/S1600536812000104

**Published:** 2012-01-11

**Authors:** Ray J. Butcher, Tapash Chakravorty, Harkesh B. Singh

**Affiliations:** a Department of Chemistry, Howard University, 525 College Street NW, Washington DC 20059, USA; b Department of Chemistry, Indian Institute of Technology Bombay, Powai, Mumbai 400 076, India

## Abstract

The asymmetric unit of the title compound, [Pd_2_(C_9_H_12_NTe)_2_Cl_2_]·0.5CH_2_Cl_2_, contains two half-mol­ecules, each lying on a twofold rotation axis; each mol­ecule is chiral and of the same enanti­omer. This is only possible as the mol­ecule has a hinged *cis* arrangement about the Pd^2+^ coordination spheres. For this hinged dimeric structure, the angles between the two coordination planes in each mol­ecule are 21.59 (4) and 22.10 (4)°. This hinged *cis* arrangement also allows the two mol­ecules to form pairs linked by secondary inter­actions between the Pd and Te atoms of an adjoining mol­ecule, leading to a tetra­meric overall structure. C—H⋯Cl inter­actions consolidate the crystal packing.

## Related literature

For related structures of bridged dimers of palladium mediated by Se, see: Brown & Corrigan (2004 ▶); Chakraborty *et al.* (2011 ▶); Dey *et al.* (2006 ▶); Ford *et al.* (2004 ▶); Kaur *et al.* (2009 ▶); Morley *et al.* (2006 ▶); Nakata *et al.* (2009 ▶); Oilunkaniemi *et al.* (1999 ▶, 2001 ▶). For Se/Te-bridged Pd dimeric structures which exhibit either a hinged or *cis* arrangement of ligands about the bridging plane, see: Kaur *et al.* (2009 ▶); Oilunkaniemi *et al.* (2000 ▶); Chakravorty *et al.* (2012 ▶). For the synthesis of the title compound, see: Chakraborty *et al.* (2011) ▶.
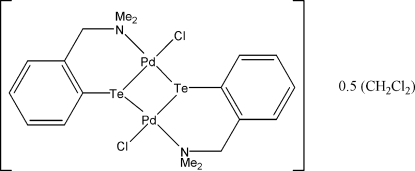


## Experimental

### 

#### Crystal data

[Pd_2_(C_9_H_12_NTe)_2_Cl_2_]·0.5CH_2_Cl_2_
*M* *_r_* = 1699.51
Orthorhombic, 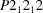

*a* = 14.035 (2) Å
*b* = 14.842 (2) Å
*c* = 12.3188 (16) Å
*V* = 2566.0 (6) Å^3^
*Z* = 2
Mo *K*α radiation
μ = 3.95 mm^−1^
*T* = 100 K
0.32 × 0.26 × 0.18 mm

#### Data collection

Bruker APEXII CCD area-detector diffractometer
Absorption correction: multi-scan (*SADABS*; Sheldrick, 1996 ▶) *T*_min_ = 0.615, *T*_max_ = 0.746
36521 measured reflections
5506 independent reflections
5148 reflections with *I* > 2σ(*I*)
*R*_int_ = 0.050

#### Refinement

*R*[*F* ^2^ > 2σ(*F* ^2^)] = 0.039
*wR*(*F* ^2^) = 0.102
*S* = 1.06
5506 reflections
254 parameters
H-atom parameters constrained
Δρ_max_ = 2.12 e Å^−3^
Δρ_min_ = −0.90 e Å^−3^
Absolute structure: Flack (1983 ▶), 2355 Friedel pairs
Flack parameter: 0.06 (4)

### 

Data collection: *APEX2* (Bruker, 2005 ▶); cell refinement: *SAINT* (Bruker, 2002 ▶); data reduction: *SAINT*; program(s) used to solve structure: *SHELXS97* (Sheldrick, 2008 ▶); program(s) used to refine structure: *SHELXL97* (Sheldrick, 2008 ▶); molecular graphics: *SHELXTL* (Sheldrick, 2008 ▶); software used to prepare material for publication: *SHELXTL*.

## Supplementary Material

Crystal structure: contains datablock(s) I, global. DOI: 10.1107/S1600536812000104/jj2116sup1.cif

Structure factors: contains datablock(s) I. DOI: 10.1107/S1600536812000104/jj2116Isup2.hkl

Additional supplementary materials:  crystallographic information; 3D view; checkCIF report

## Figures and Tables

**Table 1:** Hydrogen-bond geometry (Å, °)

*D*—H⋯*A*	*D*—H	H⋯*A*	*D*⋯*A*	*D*—H⋯*A*
C1*S*—H1*SA*⋯Cl1*B*	0.96	2.87	3.827 (9)	173
C5*A*—H5*AA*⋯Cl1*A*^i^	0.95	2.91	3.778 (9)	152
C7*A*—H7*AA*⋯Cl1*A*^i^	0.99	2.73	3.681 (9)	162
C9*A*—H9*AC*⋯Cl1*A*	0.98	2.70	3.313 (10)	121
C7*B*—H7*BA*⋯Cl1*B*^ii^	0.99	2.77	3.746 (10)	169
C7*B*—H7*BB*⋯Cl1*S*^ii^	0.99	2.75	3.514 (10)	135
C9*B*—H9*BB*⋯Cl1*B*	0.98	2.67	3.300 (11)	123

Symmetry codes: (i) 
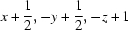
; (ii) 
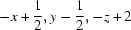
.

**Fig. 1.**
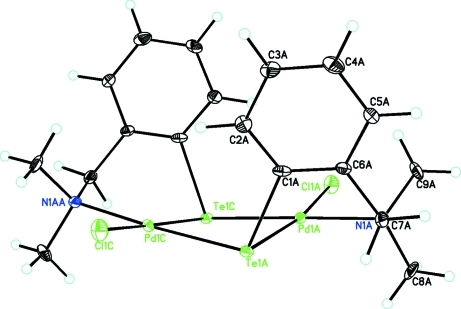
The structure of one of the two molecules of the asymmetric unit showing the hinged cis arrangement of the two Pd coordination planes. The two halves of the molecule are related by 1 - x, 1 - y, z.

**Fig. 2.**
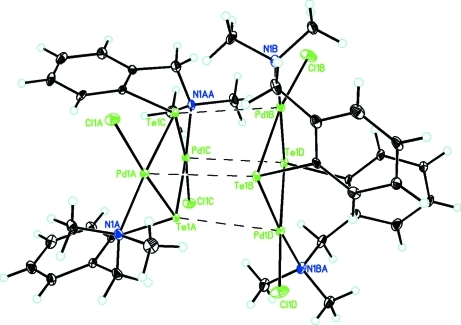
Shows the association of two dimeric units into a tetramer via matching and complementary secondary interactions between the Pd and Te of adjoining units. These interactions are shown by dashed lines.

**Fig. 3.**
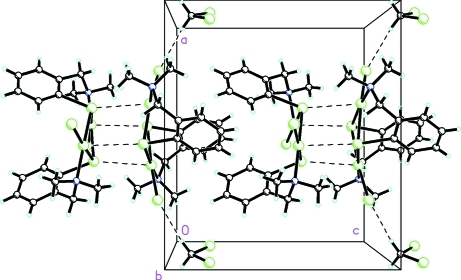
Packing diagram of the title compound viewed along the b axis. Te—Pd and C—H···Cl secondary interactions shown by dashed lines.

## supplementary crystallographic information

### Comment

The coordination chemistry of transition metal complexes with both organoselenato and organotellurato ligands is a rapidly growing area due to the ability of the resulting complexes to find applications in materials science (Morley *et al.*, 2006; Ford *et al.*, 2004), and investigations of oxidation additive to low valent transition metal centers. In addition to this, organotellurium compounds have been used in catalytic carbon-carbon formation. Bridged dimers of palladium mediated by Se (Nakata *et al.*, 2009; Chakraborty *et al.*, 2011; Oilunkaniemi *et al.*, 1999; Oilunkaniemi *et al.*, 2001; Brown & Corrigan, 2004; Dey *et al.*, 2006,) or Te (Oilunkaniemi *et al.*, 2000; Kaur *et al.*, 2009; Dey *et al.*, 2006; Chakravorty *et al.*, 2012) have been previously reported. Such dimers involving two square planar coordination spheres can adopt either a coplanar or hinged arrangement. The arrangement of the donor ligands with respect to the bridging plane can be *cis* or *trans*. In the case of a hinged *cis* arrangement the possibility of chirality exists. While the majority of previously determined Se/Te bridged Pd dimeric structures are both coplanar and *trans*, there have been a small number which exhibit either a hinged or *cis* arrangement of ligands about the bridging plane (Kaur *et al.*, 2009; Oilunkaniemi *et al.*, 2000, Chakravorty *et al.*, 2012). Of these, only that by Chakravorty *et al.*, 2012, which is the Se analog of the title complex, has resulted in a chiral complex.

The title compound, bis[µ-2-tellurolatobenzyldimethylaminochloropalladium(II)], hemi(dichloromethane) solvate, C_18_H_24_Cl_2_N_2_Pd_2_Te_2_ 0.5(CHCl_2_), crystallizes in the chiral orthorhombic space group, *P*2_1_2_1_2. The asymmetric unit contains 2 half molecules, each lying on a 2-fold axis and each molecule is chiral and of the same enantiomer. This is only possible as the molecule has a hinged *cis* arrangement about the Pd coordination spheres. For this hinged dimeric structure the angles between the two coordination planes in each molecule are 21.59 (4) and 22.10 (4)° respectively. This hinged *cis* arrangement also allows the two molecules to form pairs linked by secondary interactions between the Pd and Te of an adjoining molecule leading to a tetrameric overall structure. This hinged *cis* arrangement also allows the two molecules to form pairs linked by secondary interactions between the Pd and Te of an adjoining molecule (Fig. 2) leading to a tetrameric overall structure. Apart from this the Pd—Te, Pd—Cl and Pd—N bond lengths are in the normal ranges.

A previous polymorph of the title compound has been previously published (Kaur *et al.*, 2009). While this crystallized in the non-centrosymmetric space group, P-42_1_c, it did not result in an enantiomerically pure compound as the symmetry of the space group generated the other enantiomer. Thus this is the first example of a chiral dimeric tellurium bridged palladium compound to be structurally characterized. In both instances, however, the asymmetric unit is chiral. We believe that it is the desire of the dimers to associate which then requries the molecule to adopt the *cis* hinged structure which has lead to this inherent chirality.

### Experimental

The ligand and complex were prepared using previously reported methods (Chakraborty *et al.*, 2011). The reaction time for the synthesis of the tellurolate complex was 2 h and it was crystallized from chloroform/hexane as reported earlier. However, when the reaction was run for 30 min following the reported procedure and crystallized from dichloromethane/hexane (2:1) at ambient temperature the complex crystallized in a different space group which is chiral.

### Refinement

H atoms were placed in geometrically idealized positions and constrained to ride on their parent atoms with C—H distances of 0.95 - 0.97 Å [*U*_iso_(H) = 1.2*U*_eq_(CH, CH_2_) [*U*_iso_(H) = 1.5*U*_eq_(CH_3_)].

### Crystal data

**Table:**

[Pd_2_(C_9_H_12_NTe)_2_Cl_2_]·0.5CH_2_Cl_2_	*F*(000) = 1588
*M**_r_* = 1699.51	*D*_x_ = 2.200 Mg m^−^^3^
Orthorhombic, *P*2_1_2_1_2	Mo *K*α radiation, λ = 0.71073 Å
Hall symbol: P 2 2ab	Cell parameters from 9437 reflections
*a* = 14.035 (2) Å	θ = 2.7–26.9°
*b* = 14.842 (2) Å	µ = 3.95 mm^−^^1^
*c* = 12.3188 (16) Å	*T* = 100 K
*V* = 2566.0 (6) Å^3^	Prism, yellow-orange
*Z* = 2	0.32 × 0.26 × 0.18 mm

### Data collection

**Table:**

Bruker APEXII CCD area-detector diffractometer	5506 independent reflections
Radiation source: fine-focus sealed tube	5148 reflections with *I* > 2σ(*I*)
graphite	*R*_int_ = 0.050
ω scans	θ_max_ = 27.0°, θ_min_ = 3.1°
Absorption correction: multi-scan (*SADABS*; Sheldrick, 1996)	*h* = −17→17
*T*_min_ = 0.615, *T*_max_ = 0.746	*k* = −18→18
36521 measured reflections	*l* = −15→11

### Refinement

**Table:**

Refinement on *F*^2^	Secondary atom site location: difference Fourier map
Least-squares matrix: full	Hydrogen site location: inferred from neighbouring sites
*R*[*F*^2^ > 2σ(*F*^2^)] = 0.039	H-atom parameters constrained
*wR*(*F*^2^) = 0.102	*w* = 1/[σ^2^(*F*_o_^2^) + (0.0501*P*)^2^ + 22.7234*P*] where *P* = (*F*_o_^2^ + 2*F*_c_^2^)/3
*S* = 1.06	(Δ/σ)_max_ = 0.001
5506 reflections	Δρ_max_ = 2.12 e Å^−^^3^
254 parameters	Δρ_min_ = −0.90 e Å^−^^3^
0 restraints	Absolute structure: Flack (1983), 2355 Friedel pairs
Primary atom site location: structure-invariant direct methods	Flack parameter: 0.06 (4)

### Special details

**Table:**

Geometry. All e.s.d.'s (except the e.s.d. in the dihedral angle between two l.s. planes) are estimated using the full covariance matrix. The cell e.s.d.'s are taken into account individually in the estimation of e.s.d.'s in distances, angles and torsion angles; correlations between e.s.d.'s in cell parameters are only used when they are defined by crystal symmetry. An approximate (isotropic) treatment of cell e.s.d.'s is used for estimating e.s.d.'s involving l.s. planes.
Refinement. Refinement of *F*^2^ against ALL reflections. The weighted *R*-factor *wR* and goodness of fit *S* are based on *F*^2^, conventional *R*-factors *R* are based on *F*, with *F* set to zero for negative *F*^2^. The threshold expression of *F*^2^ > σ(*F*^2^) is used only for calculating *R*-factors(gt) *etc*. and is not relevant to the choice of reflections for refinement. *R*-factors based on *F*^2^ are statistically about twice as large as those based on *F*, and *R*- factors based on ALL data will be even larger.

### Fractional atomic coordinates and isotropic or equivalent isotropic displacement parameters (Å^2^)

**Table:**

	*x*	*y*	*z*	*U*_iso_*/*U*_eq_	
Pd1A	0.54346 (4)	0.37839 (4)	0.59921 (5)	0.01520 (13)	
Te1A	0.61163 (3)	0.53491 (4)	0.62387 (4)	0.01511 (12)	
Cl1A	0.45795 (17)	0.24470 (15)	0.5665 (2)	0.0284 (5)	
N1A	0.6857 (5)	0.3197 (5)	0.5763 (6)	0.0184 (15)	
C1A	0.6689 (6)	0.5129 (6)	0.4639 (6)	0.0177 (18)	
C2A	0.6399 (6)	0.5658 (6)	0.3784 (7)	0.0206 (17)	
H2AA	0.5937	0.6120	0.3876	0.025*	
C3A	0.6813 (7)	0.5486 (6)	0.2777 (7)	0.025 (2)	
H3AA	0.6611	0.5822	0.2163	0.030*	
C4A	0.7500 (7)	0.4848 (6)	0.2654 (8)	0.028 (2)	
H4AA	0.7786	0.4756	0.1964	0.034*	
C5A	0.7784 (6)	0.4330 (6)	0.3537 (7)	0.0226 (18)	
H5AA	0.8267	0.3886	0.3456	0.027*	
C6A	0.7354 (6)	0.4470 (6)	0.4537 (6)	0.0169 (17)	
C7A	0.7615 (6)	0.3882 (7)	0.5500 (7)	0.0237 (19)	
H7AA	0.8219	0.3565	0.5341	0.028*	
H7AB	0.7721	0.4270	0.6142	0.028*	
C8A	0.7109 (7)	0.2750 (7)	0.6799 (8)	0.032 (2)	
H8AA	0.7769	0.2536	0.6764	0.047*	
H8AB	0.6681	0.2238	0.6921	0.047*	
H8AC	0.7044	0.3181	0.7397	0.047*	
C9A	0.6846 (7)	0.2495 (7)	0.4917 (8)	0.027 (2)	
H9AA	0.7483	0.2230	0.4850	0.040*	
H9AB	0.6660	0.2763	0.4222	0.040*	
H9AC	0.6389	0.2024	0.5116	0.040*	
Pd1B	0.37084 (4)	0.45871 (4)	0.90101 (5)	0.01594 (14)	
Te1B	0.53688 (4)	0.39525 (3)	0.87669 (4)	0.01555 (12)	
Cl1B	0.22964 (17)	0.54079 (18)	0.9375 (2)	0.0370 (6)	
N1B	0.3072 (5)	0.3220 (5)	0.9235 (6)	0.0207 (15)	
C1B	0.5141 (6)	0.3441 (6)	1.0358 (6)	0.0175 (17)	
C2B	0.5672 (6)	0.3731 (6)	1.1223 (7)	0.0244 (19)	
H2BA	0.6144	0.4182	1.1127	0.029*	
C3B	0.5510 (7)	0.3351 (7)	1.2270 (7)	0.026 (2)	
H3BA	0.5845	0.3568	1.2887	0.032*	
C4B	0.4853 (6)	0.2656 (6)	1.2371 (7)	0.025 (2)	
H4BA	0.4775	0.2366	1.3052	0.030*	
C5B	0.4308 (7)	0.2378 (7)	1.1491 (8)	0.030 (2)	
H5BA	0.3850	0.1913	1.1583	0.035*	
C6B	0.4430 (6)	0.2783 (6)	1.0454 (7)	0.0217 (18)	
C7B	0.3803 (7)	0.2521 (6)	0.9532 (7)	0.025 (2)	
H7BA	0.3471	0.1954	0.9721	0.030*	
H7BB	0.4206	0.2400	0.8889	0.030*	
C8B	0.2650 (8)	0.2964 (7)	0.8194 (7)	0.029 (2)	
H8BA	0.2382	0.2356	0.8250	0.043*	
H8BB	0.3142	0.2975	0.7630	0.043*	
H8BC	0.2143	0.3391	0.8007	0.043*	
C9B	0.2331 (7)	0.3257 (7)	1.0054 (7)	0.025 (2)	
H9BA	0.2028	0.2665	1.0119	0.038*	
H9BB	0.1853	0.3705	0.9844	0.038*	
H9BC	0.2612	0.3427	1.0753	0.038*	
Cl1S	−0.0241 (2)	0.5950 (2)	1.1834 (4)	0.0643 (10)	
C1S	0.0000	0.5000	1.0976 (13)	0.044 (4)	
H1SA	0.0542	0.5111	1.0521	0.052*	

### Atomic displacement parameters (Å^2^)

**Table:**

	*U*^11^	*U*^22^	*U*^33^	*U*^12^	*U*^13^	*U*^23^
Pd1A	0.0164 (3)	0.0155 (3)	0.0137 (3)	0.0010 (2)	−0.0010 (2)	0.0024 (2)
Te1A	0.0151 (2)	0.0183 (2)	0.0119 (2)	−0.00024 (19)	0.00035 (18)	−0.0012 (2)
Cl1A	0.0258 (10)	0.0191 (10)	0.0401 (12)	−0.0044 (9)	−0.0013 (10)	−0.0009 (9)
N1A	0.019 (3)	0.023 (4)	0.013 (3)	0.007 (3)	0.000 (3)	0.001 (3)
C1A	0.018 (4)	0.026 (5)	0.009 (3)	−0.007 (3)	0.001 (3)	−0.001 (3)
C2A	0.023 (4)	0.017 (4)	0.021 (4)	−0.004 (3)	0.007 (4)	0.005 (3)
C3A	0.041 (5)	0.014 (4)	0.020 (4)	−0.008 (4)	0.000 (4)	0.007 (3)
C4A	0.033 (5)	0.031 (5)	0.021 (4)	−0.006 (4)	0.010 (4)	−0.001 (4)
C5A	0.026 (4)	0.023 (4)	0.019 (4)	0.005 (4)	0.002 (4)	−0.004 (3)
C6A	0.017 (4)	0.022 (5)	0.012 (4)	−0.001 (3)	0.000 (3)	−0.003 (3)
C7A	0.020 (4)	0.031 (5)	0.020 (4)	0.011 (4)	0.002 (3)	−0.001 (4)
C8A	0.033 (5)	0.035 (6)	0.027 (5)	0.016 (4)	−0.007 (4)	0.014 (4)
C9A	0.032 (5)	0.020 (4)	0.028 (5)	0.008 (4)	0.007 (4)	−0.009 (4)
Pd1B	0.0185 (3)	0.0145 (3)	0.0147 (3)	−0.0018 (2)	0.0007 (2)	−0.0024 (2)
Te1B	0.0207 (2)	0.0142 (2)	0.0117 (2)	−0.0004 (2)	0.0012 (2)	0.00052 (19)
Cl1B	0.0274 (11)	0.0253 (12)	0.0584 (16)	0.0041 (10)	0.0144 (11)	−0.0036 (12)
N1B	0.029 (4)	0.017 (4)	0.016 (3)	−0.003 (3)	0.004 (3)	−0.001 (3)
C1B	0.016 (4)	0.028 (4)	0.009 (3)	0.002 (3)	0.003 (3)	0.005 (3)
C2B	0.021 (4)	0.035 (5)	0.017 (4)	0.006 (4)	0.000 (3)	0.020 (4)
C3B	0.022 (4)	0.042 (5)	0.015 (4)	−0.001 (4)	−0.001 (4)	0.005 (4)
C4B	0.025 (5)	0.034 (5)	0.015 (4)	0.008 (4)	0.006 (3)	0.013 (4)
C5B	0.030 (5)	0.031 (5)	0.027 (5)	0.002 (4)	0.005 (4)	0.012 (4)
C6B	0.024 (5)	0.021 (4)	0.020 (4)	0.006 (4)	0.004 (3)	0.002 (3)
C7B	0.036 (5)	0.023 (5)	0.018 (4)	0.004 (4)	0.011 (4)	0.003 (4)
C8B	0.042 (6)	0.027 (5)	0.018 (4)	−0.023 (4)	−0.002 (4)	−0.004 (4)
C9B	0.028 (5)	0.028 (5)	0.019 (4)	−0.002 (4)	0.007 (4)	−0.002 (4)
Cl1S	0.0516 (19)	0.0396 (15)	0.102 (3)	−0.0078 (15)	0.0240 (18)	0.0024 (18)
C1S	0.034 (7)	0.062 (10)	0.034 (8)	0.002 (7)	0.000	0.000

### Geometric parameters (Å, °)

**Table:**

Pd1A—N1A	2.196 (7)	Pd1B—Te1B	2.5313 (8)
Pd1A—Cl1A	2.354 (2)	Pd1B—Te1B^i^	2.5427 (8)
Pd1A—Te1A	2.5305 (8)	Pd1B—Te1A^i^	3.4241 (9)
Pd1A—Te1A^i^	2.5467 (8)	Te1B—C1B	2.126 (8)
Pd1A—Te1B	3.4286 (9)	Te1B—Pd1B^i^	2.5427 (8)
Te1A—C1A	2.154 (8)	N1B—C9B	1.449 (11)
Te1A—Pd1A^i^	2.5467 (8)	N1B—C8B	1.462 (11)
N1A—C9A	1.474 (12)	N1B—C7B	1.504 (12)
N1A—C8A	1.480 (11)	C1B—C2B	1.370 (12)
N1A—C7A	1.508 (12)	C1B—C6B	1.402 (13)
C1A—C6A	1.357 (13)	C2B—C3B	1.426 (11)
C1A—C2A	1.375 (12)	C2B—H2BA	0.9500
C2A—C3A	1.393 (12)	C3B—C4B	1.389 (13)
C2A—H2AA	0.9500	C3B—H3BA	0.9500
C3A—C4A	1.360 (13)	C4B—C5B	1.389 (14)
C3A—H3AA	0.9500	C4B—H4BA	0.9500
C4A—C5A	1.390 (13)	C5B—C6B	1.422 (12)
C4A—H4AA	0.9500	C5B—H5BA	0.9500
C5A—C6A	1.388 (11)	C6B—C7B	1.488 (13)
C5A—H5AA	0.9500	C7B—H7BA	0.9900
C6A—C7A	1.517 (12)	C7B—H7BB	0.9900
C7A—H7AA	0.9900	C8B—H8BA	0.9800
C7A—H7AB	0.9900	C8B—H8BB	0.9800
C8A—H8AA	0.9800	C8B—H8BC	0.9800
C8A—H8AB	0.9800	C9B—H9BA	0.9800
C8A—H8AC	0.9800	C9B—H9BB	0.9800
C9A—H9AA	0.9800	C9B—H9BC	0.9800
C9A—H9AB	0.9800	Cl1S—C1S	1.794 (10)
C9A—H9AC	0.9800	C1S—Cl1S^ii^	1.794 (10)
Pd1B—N1B	2.235 (7)	C1S—H1SA	0.9600
Pd1B—Cl1B	2.369 (2)		
			
N1A—Pd1A—Cl1A	96.1 (2)	N1B—Pd1B—Te1B^i^	172.9 (2)
N1A—Pd1A—Te1A	92.1 (2)	Cl1B—Pd1B—Te1B^i^	90.58 (7)
Cl1A—Pd1A—Te1A	170.82 (7)	Te1B—Pd1B—Te1B^i^	80.46 (3)
N1A—Pd1A—Te1A^i^	173.0 (2)	N1B—Pd1B—Te1A^i^	100.24 (19)
Cl1A—Pd1A—Te1A^i^	90.61 (6)	Cl1B—Pd1B—Te1A^i^	103.60 (7)
Te1A—Pd1A—Te1A^i^	81.08 (2)	Te1B—Pd1B—Te1A^i^	79.99 (2)
N1A—Pd1A—Te1B	100.45 (18)	Te1B^i^—Pd1B—Te1A^i^	79.77 (2)
Cl1A—Pd1A—Te1B	102.63 (6)	C1B—Te1B—Pd1B	83.4 (2)
Te1A—Pd1A—Te1B	79.84 (2)	C1B—Te1B—Pd1B^i^	105.8 (2)
Te1A^i^—Pd1A—Te1B	79.70 (2)	Pd1B—Te1B—Pd1B^i^	97.92 (3)
C1A—Te1A—Pd1A	83.8 (2)	C1B—Te1B—Pd1A	153.8 (2)
C1A—Te1A—Pd1A^i^	106.7 (2)	Pd1B—Te1B—Pd1A	99.78 (2)
Pd1A—Te1A—Pd1A^i^	97.26 (3)	Pd1B^i^—Te1B—Pd1A	99.55 (2)
C9A—N1A—C8A	107.2 (7)	C9B—N1B—C8B	109.2 (8)
C9A—N1A—C7A	109.4 (7)	C9B—N1B—C7B	110.3 (7)
C8A—N1A—C7A	108.6 (7)	C8B—N1B—C7B	108.1 (7)
C9A—N1A—Pd1A	111.2 (6)	C9B—N1B—Pd1B	109.8 (6)
C8A—N1A—Pd1A	106.5 (5)	C8B—N1B—Pd1B	106.8 (5)
C7A—N1A—Pd1A	113.7 (5)	C7B—N1B—Pd1B	112.5 (6)
C6A—C1A—C2A	123.0 (8)	C2B—C1B—C6B	122.7 (8)
C6A—C1A—Te1A	116.8 (6)	C2B—C1B—Te1B	121.6 (6)
C2A—C1A—Te1A	120.2 (6)	C6B—C1B—Te1B	115.7 (6)
C1A—C2A—C3A	116.9 (8)	C1B—C2B—C3B	119.5 (8)
C1A—C2A—H2AA	121.5	C1B—C2B—H2BA	120.2
C3A—C2A—H2AA	121.5	C3B—C2B—H2BA	120.2
C4A—C3A—C2A	121.5 (9)	C4B—C3B—C2B	118.7 (9)
C4A—C3A—H3AA	119.2	C4B—C3B—H3BA	120.6
C2A—C3A—H3AA	119.2	C2B—C3B—H3BA	120.6
C3A—C4A—C5A	120.1 (9)	C5B—C4B—C3B	121.1 (8)
C3A—C4A—H4AA	120.0	C5B—C4B—H4BA	119.4
C5A—C4A—H4AA	120.0	C3B—C4B—H4BA	119.4
C6A—C5A—C4A	119.1 (8)	C4B—C5B—C6B	120.6 (9)
C6A—C5A—H5AA	120.5	C4B—C5B—H5BA	119.7
C4A—C5A—H5AA	120.5	C6B—C5B—H5BA	119.7
C1A—C6A—C5A	119.3 (8)	C1B—C6B—C5B	117.2 (8)
C1A—C6A—C7A	120.5 (7)	C1B—C6B—C7B	122.5 (8)
C5A—C6A—C7A	120.1 (8)	C5B—C6B—C7B	120.3 (8)
N1A—C7A—C6A	112.7 (7)	C6B—C7B—N1B	114.1 (7)
N1A—C7A—H7AA	109.0	C6B—C7B—H7BA	108.7
C6A—C7A—H7AA	109.0	N1B—C7B—H7BA	108.7
N1A—C7A—H7AB	109.0	C6B—C7B—H7BB	108.7
C6A—C7A—H7AB	109.0	N1B—C7B—H7BB	108.7
H7AA—C7A—H7AB	107.8	H7BA—C7B—H7BB	107.6
N1A—C8A—H8AA	109.5	N1B—C8B—H8BA	109.5
N1A—C8A—H8AB	109.5	N1B—C8B—H8BB	109.5
H8AA—C8A—H8AB	109.5	H8BA—C8B—H8BB	109.5
N1A—C8A—H8AC	109.5	N1B—C8B—H8BC	109.5
H8AA—C8A—H8AC	109.5	H8BA—C8B—H8BC	109.5
H8AB—C8A—H8AC	109.5	H8BB—C8B—H8BC	109.5
N1A—C9A—H9AA	109.5	N1B—C9B—H9BA	109.5
N1A—C9A—H9AB	109.5	N1B—C9B—H9BB	109.5
H9AA—C9A—H9AB	109.5	H9BA—C9B—H9BB	109.5
N1A—C9A—H9AC	109.5	N1B—C9B—H9BC	109.5
H9AA—C9A—H9AC	109.5	H9BA—C9B—H9BC	109.5
H9AB—C9A—H9AC	109.5	H9BB—C9B—H9BC	109.5
N1B—Pd1B—Cl1B	96.3 (2)	Cl1S—C1S—Cl1S^ii^	107.8 (9)
N1B—Pd1B—Te1B	92.6 (2)	Cl1S—C1S—H1SA	111.0
Cl1B—Pd1B—Te1B	169.67 (7)	Cl1S^ii^—C1S—H1SA	109.2
			
N1A—Pd1A—Te1A—C1A	58.9 (3)	Cl1B—Pd1B—Te1B—Pd1A	−117.7 (4)
Cl1A—Pd1A—Te1A—C1A	−94.4 (5)	Te1B^i^—Pd1B—Te1B—Pd1A	−87.59 (2)
Te1A^i^—Pd1A—Te1A—C1A	−119.8 (2)	Te1A^i^—Pd1B—Te1B—Pd1A	−6.410 (18)
Te1B—Pd1A—Te1A—C1A	159.2 (2)	N1A—Pd1A—Te1B—C1B	−83.5 (5)
N1A—Pd1A—Te1A—Pd1A^i^	164.97 (18)	Cl1A—Pd1A—Te1B—C1B	15.3 (5)
Cl1A—Pd1A—Te1A—Pd1A^i^	11.6 (4)	Te1A—Pd1A—Te1B—C1B	−173.7 (5)
Te1A^i^—Pd1A—Te1A—Pd1A^i^	−13.74 (4)	Te1A^i^—Pd1A—Te1B—C1B	103.6 (5)
Te1B—Pd1A—Te1A—Pd1A^i^	−94.78 (2)	N1A—Pd1A—Te1B—Pd1B	−178.4 (2)
Cl1A—Pd1A—N1A—C9A	34.8 (6)	Cl1A—Pd1A—Te1B—Pd1B	−79.66 (7)
Te1A—Pd1A—N1A—C9A	−141.0 (6)	Te1A—Pd1A—Te1B—Pd1B	91.32 (3)
Te1A^i^—Pd1A—N1A—C9A	−130.5 (14)	Te1A^i^—Pd1A—Te1B—Pd1B	8.64 (2)
Te1B—Pd1A—N1A—C9A	138.9 (6)	N1A—Pd1A—Te1B—Pd1B^i^	81.7 (2)
Cl1A—Pd1A—N1A—C8A	−81.7 (6)	Cl1A—Pd1A—Te1B—Pd1B^i^	−179.49 (6)
Te1A—Pd1A—N1A—C8A	102.5 (6)	Te1A—Pd1A—Te1B—Pd1B^i^	−8.52 (2)
Te1A^i^—Pd1A—N1A—C8A	113.0 (15)	Te1A^i^—Pd1A—Te1B—Pd1B^i^	−91.20 (3)
Te1B—Pd1A—N1A—C8A	22.4 (6)	Cl1B—Pd1B—N1B—C9B	−32.5 (6)
Cl1A—Pd1A—N1A—C7A	158.8 (5)	Te1B—Pd1B—N1B—C9B	142.1 (6)
Te1A—Pd1A—N1A—C7A	−17.1 (5)	Te1B^i^—Pd1B—N1B—C9B	133.0 (14)
Te1A^i^—Pd1A—N1A—C7A	−6.5 (19)	Te1A^i^—Pd1B—N1B—C9B	−137.6 (6)
Te1B—Pd1A—N1A—C7A	−97.1 (5)	Cl1B—Pd1B—N1B—C8B	85.8 (6)
Pd1A—Te1A—C1A—C6A	−65.8 (6)	Te1B—Pd1B—N1B—C8B	−99.6 (6)
Pd1A^i^—Te1A—C1A—C6A	−161.5 (6)	Te1B^i^—Pd1B—N1B—C8B	−108.7 (15)
Pd1A—Te1A—C1A—C2A	115.9 (7)	Te1A^i^—Pd1B—N1B—C8B	−19.3 (6)
Pd1A^i^—Te1A—C1A—C2A	20.1 (7)	Cl1B—Pd1B—N1B—C7B	−155.8 (5)
C6A—C1A—C2A—C3A	0.7 (13)	Te1B—Pd1B—N1B—C7B	18.8 (5)
Te1A—C1A—C2A—C3A	178.9 (6)	Te1B^i^—Pd1B—N1B—C7B	9.7 (19)
C1A—C2A—C3A—C4A	−2.5 (13)	Te1A^i^—Pd1B—N1B—C7B	99.1 (5)
C2A—C3A—C4A—C5A	2.0 (14)	Pd1B—Te1B—C1B—C2B	−114.6 (7)
C3A—C4A—C5A—C6A	0.4 (14)	Pd1B^i^—Te1B—C1B—C2B	−18.2 (8)
C2A—C1A—C6A—C5A	1.6 (13)	Pd1A—Te1B—C1B—C2B	146.7 (6)
Te1A—C1A—C6A—C5A	−176.7 (6)	Pd1B—Te1B—C1B—C6B	66.6 (6)
C2A—C1A—C6A—C7A	−177.4 (8)	Pd1B^i^—Te1B—C1B—C6B	163.0 (6)
Te1A—C1A—C6A—C7A	4.3 (11)	Pd1A—Te1B—C1B—C6B	−32.2 (10)
C4A—C5A—C6A—C1A	−2.2 (13)	C6B—C1B—C2B—C3B	0.5 (14)
C4A—C5A—C6A—C7A	176.9 (8)	Te1B—C1B—C2B—C3B	−178.3 (7)
C9A—N1A—C7A—C6A	71.5 (9)	C1B—C2B—C3B—C4B	3.7 (14)
C8A—N1A—C7A—C6A	−171.9 (7)	C2B—C3B—C4B—C5B	−4.8 (14)
Pd1A—N1A—C7A—C6A	−53.5 (8)	C3B—C4B—C5B—C6B	1.8 (14)
C1A—C6A—C7A—N1A	74.3 (10)	C2B—C1B—C6B—C5B	−3.4 (13)
C5A—C6A—C7A—N1A	−104.7 (9)	Te1B—C1B—C6B—C5B	175.4 (7)
N1B—Pd1B—Te1B—C1B	−60.2 (3)	C2B—C1B—C6B—C7B	175.1 (8)
Cl1B—Pd1B—Te1B—C1B	88.6 (5)	Te1B—C1B—C6B—C7B	−6.1 (11)
Te1B^i^—Pd1B—Te1B—C1B	118.7 (2)	C4B—C5B—C6B—C1B	2.3 (13)
Te1A^i^—Pd1B—Te1B—C1B	−160.1 (2)	C4B—C5B—C6B—C7B	−176.2 (8)
N1B—Pd1B—Te1B—Pd1B^i^	−165.28 (19)	C1B—C6B—C7B—N1B	−72.2 (11)
Cl1B—Pd1B—Te1B—Pd1B^i^	−16.5 (4)	C5B—C6B—C7B—N1B	106.3 (10)
Te1B^i^—Pd1B—Te1B—Pd1B^i^	13.59 (4)	C9B—N1B—C7B—C6B	−72.4 (10)
Te1A^i^—Pd1B—Te1B—Pd1B^i^	94.77 (2)	C8B—N1B—C7B—C6B	168.3 (7)
N1B—Pd1B—Te1B—Pd1A	93.54 (19)	Pd1B—N1B—C7B—C6B	50.6 (8)

Symmetry codes: (i) −*x*+1, −*y*+1, *z*; (ii) −*x*, −*y*+1, *z*.

### Hydrogen-bond geometry (Å, °)

**Table:**

*D*—H···*A*	*D*—H	H···*A*	*D*···*A*	*D*—H···*A*
C1S—H1SA···Cl1B	0.96	2.87	3.827 (9)	173
C5A—H5AA···Cl1A^iii^	0.95	2.91	3.778 (9)	152
C7A—H7AA···Cl1A^iii^	0.99	2.73	3.681 (9)	162
C9A—H9AC···Cl1A	0.98	2.70	3.313 (10)	121
C7B—H7BA···Cl1B^iv^	0.99	2.77	3.746 (10)	169
C7B—H7BB···Cl1S^iv^	0.99	2.75	3.514 (10)	135
C9B—H9BB···Cl1B	0.98	2.67	3.300 (11)	123

Symmetry codes: (iii) *x*+1/2, −*y*+1/2, −*z*+1; (iv) −*x*+1/2, *y*−1/2, −*z*+2.
